# The Casket and the Ribbon, or the Honors of Ether

**Published:** 1849-04

**Authors:** 


*The Casket and the Ribbon, or the Honors of Ether,* from the Cazenovia editor, was not received until all but the last form of the present number of the Journal was in type. It will appear in the next number.

[*Balt. Ed.*

